# Food and nutrient gaps in rural Northern Ghana: Does production of smallholder farming households support adoption of food-based dietary guidelines?

**DOI:** 10.1371/journal.pone.0204014

**Published:** 2018-09-13

**Authors:** Ilse de Jager, Ken E. Giller, Inge D. Brouwer

**Affiliations:** 1 Division of Human Nutrition, Wageningen University, Wageningen, The Netherlands; 2 Plant Production Systems group, Wageningen University, Wageningen, The Netherlands; Institut de recherche pour le developpement, FRANCE

## Abstract

Food-based dietary guidelines (FBDGs) provide guidance to policy makers, the private sector and consumers to redesign food systems and to improve diets of vulnerable populations. As appropriate FBDGs are based on the actual dietary patterns and their costs, it is assumed that the recommended foods are available, affordable and acceptable for the population under study. Using quantitative dietary intake data of young children in rural Northern Ghana, we developed local FBDGs and studied whether these are supported by the diversity and quantity of the production of a household among 329 households. We found that 40% of rural Northern Ghanaian infants and young children were stunted and their nutrient intakes were far below the recommendations: the probability of adequacy for most nutrient intakes was less than 50%. At household level, the developed FBDGs were, on average, unable to sufficiently cover the household requirements for fat (60.4% of recommended nutrient intake (RNI)), calcium (34.3% RNI), iron (60.3% RNI), vitamin A (39.1% RNI), vitamin B12 (2.3% RNI) and vitamin C (54.6% RNI). This implies that even when these FBDGs are fully adopted the requirements for these nutrients will not be met. In addition, the nutrient needs and food needs (according to the developed FBDGs) of a household were only marginally covered by their own food production. The food production of over half the households supplied insufficient calcium (75.7%), vitamin A (100%), vitamin B12 (100%) and vitamin C (77.5%) to cover their needs. The food production of about 60% of the households did not cover their required quantities of grains and legumes and none covered their required quantities of vegetables. Further analysis of the food gaps at district and national level showed that sufficient grains were available at both levels (267% and 148%, respectively) to meet requirements; availability of legumes was sufficient at district level (268%) but not at national level (52%); and vegetables were insufficient at both levels (2% and 49%, respectively). Diversifying household food production is often proposed as a means to increase the diversity of foods available and thereby increasing dietary diversity of rural populations. We found that the diversity of the production of a household was indeed positively related with their food and nutrient coverage. However, the diversity of the production of a household and their food and nutrient coverage were not related with children’s dietary diversity and nutrient adequacy. Our results show that the production of a households does not support the adoption of FBDGs in rural Northern Ghana, especially for vegetables. This suggests that the promotion of FBDGs through nutrition education or behaviour change communications activities alone is insufficient to lead to improvements in diets. Additional strategies are needed to increase the food availability and accessibility of the households, especially that of fruits and vegetables, such as diversification of the crops grown, increased production of specific crops and market-based strategies.

## Introduction

Current transformations of food systems driven by climate change, urbanization, income growth and population growth are often associated with unhealthy diets as they fail to provide sufficient, diverse, nutritious and safe food for all [1]. Among low and middle income country (LMIC) populations the average diets fall far short of the recommended quantities of fruits, vegetables, dairy and other protein-rich foods [2]. Undernutrition persists, especially in rural areas of sub-Saharan Africa where one in three children is chronically malnourished and micronutrient deficiencies prevail [3,4]. This impairs physical and mental development resulting in a life-long disadvantage [5]. Simultaneously the number of overweight children is increasing [3]. One of the many causes of malnutrition lies in low-quality diets [1]. Malnutrition associated with low-quality diets is the number one risk factor in the global burden of disease [6]. Food-based dietary guidelines (FBDGs) provide guidance to policy makers, private sector and consumers to redesign food systems and to improve diets of vulnerable populations [1]. However, FBDGs are largely absent in LMICs and especially in Africa where only 7 out of 58 countries have official FBDGs [7].

FBDGs that provide sufficient nutrients required by LMIC populations have recently been developed using linear programming [8,9]. These studies based their analysis on actual dietary patterns and their costs–in doing so they implicitly assumed that the developed FBDGs are available, affordable and acceptable for the population under study [10]. However, their analysis is based on the distribution of the types and frequencies of foods consumed, and often uses the extreme ends of these distributions to arrive at FBDGs that cover most of the nutrient needs. Using extremes values may limit the adoption of local FBDGs as the recommended quantity of foods may not be available, affordable and/or accepted by the targeted population. It therefore remains unclear whether the developed FBDGs are supported by the local food system.

**Fig 1:**
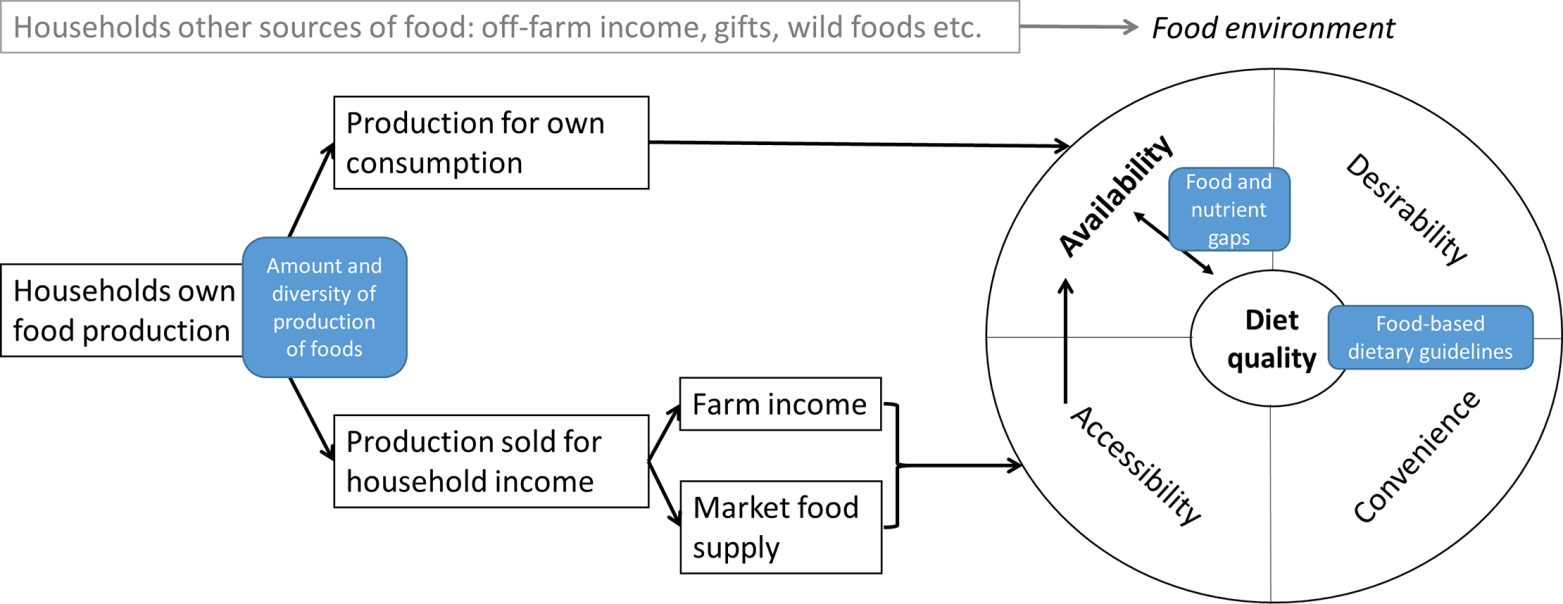
The production-own consumption and the income-food purchase pathways are two pathways that contribute to the availability and accessibility of food: A key condition for the adoption of food-based dietary guidelines to improve diet quality.

The availability of recommended foods is a key condition for the adoption of FBDGs [11] (Fig 1). Although most people in rural areas do not depend solely on their own agricultural production for their food and income, their production is often the most important source of food [12].The price farmers receive for their produce is often not enough to cover the retail price of foods that they decided not to grow. Therefore rural households tend to prefer to intensify their own production of food crops for home consumption and to sell only the surplus that is produced after all their food needs have been met [13]. In addition, many rural households have an income based mainly on the sale of their produce: in rural Northern Ghana over 80% of households reported that all or three quarters of their income was from their own food production [14]. In general, two main pathways make the production of households available for improved diets and nutrition outcomes in LMICs [15]. The first pathway refers to crop production for own consumption (the production-own consumption pathway) and assumes that increased production of nutritious foods increases consumption of these foods and adds to diversity of the diets of the household and of individuals [15]. The second pathway refers to production sold for household income and assumes that agricultural income through sale of production is used for immediate or future household needs, including food purchases to support improved dietary diversity (the income-food purchase pathway) [15]. In addition, this assumes the required foods are available at local markets. Market access may have larger positive effects on the dietary diversity of households than the diversity of the production of households [16]. Although agriculture income growth may not be sufficient to ensure improved dietary diversity, it seems to increase the share of vegetable, fish and tuber consumption [17]. Two recent reviews show that increasing diversity of crop production of smallholder households in LIMC is associated with more diverse diets at household and individual level [18,19]. Therefore, it is hypothesized that the production of households, either via the production-own consumption pathway or via the income-food purchase pathway, contributes to the diversity and quantity of foods available and accessible for household consumption and thereby determines whether and to what extent adoption of FBDGs is possible.

An understanding of whether and to what extent households can meet their food and nutrient needs through their own production and how this is associated with the quality of a children’s diet may inform what strategies are required to further facilitate adoption of FBDGs to improve diets of vulnerable groups in rural areas. To this end we used dietary intake data of young children in rural Northern Ghana to develop local FBDGs and studied whether these are supported by the quantities and diversity of foods produced at household and district level. At national level we studied whether FBDGs are supported by national food availability per capita (accounting for food imports, exports and waste). In addition, we studied whether diversifying the production of households own food production has potential to increase the diversity of foods available and accessible and thereby increasing children’s dietary diversity and nutrient adequacy.

## Materials and methods

### Study area

The study was carried out in Karaga sub-district in the Northern Region of Ghana. Northern Ghana has one cropping season that lasts 5 to 6 months starting in May, an average annual temperature of 28°C and annual rainfall of 900 to 1040 mm. The main crops in Northern Ghana are maize, rice, cowpea and yam. Travel time to urban markets is between 1 to 7 hours and population density is sparse with 50 to 100 inhabitants per km^2^ [20]. Karaga district was selected from Northern Region because of high food insecurity and malnutrition. About 32% of children below 5 years old are stunted and 9% are wasted [21].

### Study population and sampling strategy

A census was conducted in Karaga sub-district between May-June 2014 to identify all households with children of 6–23 months and collect information on their sex, date of birth, breastfeeding status and geographical location by GPS coordinates. A list of all households with children of 6–23 months in Karaga sub-district constituted the sampling frame divided into four sub-frames corresponding to the four age groups. A random order list was developed for each sub-frame and the first 100 children on this list were selected. To develop local FBDGs using linear programming software (e-Optifood), the study population was divided into four specific groups according to age and breastfeeding state: 6–8 months breastfed, 9–11 months breastfed, 12–23 months breastfed and 12–23 months non-breastfed. A household was defined as ‘a person or group of related or unrelated persons who live together in the same housing unit, sharing the same housekeeping and cooking arrangements, and who acknowledge an adult male or female as the head of the household’.

Eligibility was defined by the age of the child falling between 6–23 months using the day before the start of data collection as the reference date (30 June 2014). For the breastfed groups, eligibility was also defined as receiving both breastfeeding and complementary feeding. Eligibility for the study was cross-checked in the field prior to the start of data collection and ineligible children were randomly replaced with other eligible children in the same community or nearby community. A sample size of approximately 100 per group was determined based on estimated population mean food serving sizes for commonly consumed foods in the study area to be within 10% (95% CI), assuming an SD of 50% of the mean serving sizes in the age group and allowing for a 5% rate of attrition [22]. One child per household was selected. In case two or more children in the household qualified for inclusion, one was randomly chosen. Communities of selected children were clustered into three geographic areas: north, central and south. Each cluster was then randomly assigned to a time slot of data collection. For this study, children of households that either did not farm (*n* = 7) or had no harvest during the last year (*n* = 1) were excluded. A random sample of food vendors within the selected study communities and major markets within the study area were also interviewed to determine prices of foods identified during collection of dietary data.

### Data collection and analysis

Data was collected in Ghana in July 2014 by trained enumerators who had a first degree in nutrition and who spoke the local language. Trained supervisors with previous experience in dietary assessment and who spoke the local language observed some of the interviews and back-checked data forms of all interviews. In case of inconsistencies, the survey supervisors ensured that households were revisited. Dietary assessment was conducted with the mother or primary caretaker of the selected children. A structured questionnaire-based interview was conducted with the head of household of the selected child to collect information on household composition, education, occupation, sources of income, religion, total cultivated land, distance to closest market and available functioning assets (radio, television, bicycle, motor, corn mill, private and/or commercial vehicle). Total value of assets in each household was calculated in Ghanaian Cedi’s (GH₵) by estimated local market value and converted into purchasing power parity in US dollar using the conversion factor of 2014 of 1.032 [23]. Details on data collection and analysis can be found in supplementary material (S1 Text).

#### Children’s nutritional status

Weight and length of children were measured following standard procedures. Length and weight measurements were converted into height-for-age, weight-for-height, weight-for-age and BMI-for-age z-scores based on the WHO Child Growth Standards by using the WHO SPSS syntax. Children who were more than two SD below reference median of height-for-age, weight-for-height and weight-for-age z-scores were classified to be stunted, wasted and underweight. Children who were more than two SD above reference median of BMI-for-age were classified to be overweight.

#### Food composition table

A food composition table was specifically created for this study (sFCT) using nutrient values primarily from the West African Food Composition Table [24] and complemented with values from other sources. Energy and the following nutrients: protein, carbohydrates (by difference), fat, water, calcium, iron, zinc, vitamin A (RAE), folate, vitamin C, thiamine, riboflavin, niacin, vitamin B_6_, and vitamin B_12_ were derived.

#### Children’s dietary intake

Dietary intakes of the children were assessed using a quantitative multi-pass 24-hour recall (24hR) [25]. All days of the week were captured and randomly assigned to subjects to account for day-to-day variation in dietary intake. Data was collected within a time period of 3 weeks.

*Children’s dietary diversity*: Dietary intake data was used to calculate the individual dietary diversity score (IDDS) being a count of the number of seven different food groups consumed, including: (i) grains, roots and/or tubers; (ii) legumes and/or nuts; (iii) dairy products; (iv) flesh foods; (v) eggs; (vi) vitamin A rich fruits and/or vegetables; and (vii) other fruits and/or vegetables [26]. Consumption of any quantity of food from each food group was sufficient to ‘count’, except if an item was used as a condiment. Fruits and vegetables were classified as vitamin-A rich when they provided at least 60 retinol activity equivalents (RAE) per 100 g. Consumption of at least four out of these seven is associated with adequate dietary quality of children of 6–23 months [26]. Median IDDS and the proportion of children who had a nutrient diverse diet (IDDS = >4) were calculated.

*Children’s nutrient adequacy*: Nutrient intakes were calculated based on the sFCT and using nutrient calculation system Compl-eat^TM^ (version 1.0, Wageningen University). To generate usual intakes, nutrient intakes were adjusted for within-person variation using the National Research Council adjustment method [27,28]. For breastfed children, intake of breastmilk was not measured directly and therefore we assumed average intakes based on estimated energy intakes from breastmilk for populations in low income countries [29,30]. The total nutrient intake for breastfed children were computed by their adjusted nutrient intakes plus the nutrient intake from the assumed average breastmilk intakes [30]. Intakes of 11 key micronutrients were assessed: iron, zinc, calcium, vitamin A, vitamin C, thiamine, riboflavin, niacin, vitamin B_6_, folate, and vitamin B_12_. Except for iron, the probability of adequacy (PA) of each nutrient was calculated based on their respective estimated average requirements (EARs) and distributions [31,32] (S1 Table). EAR represents the quantity of a nutrient that ensures the needs of 50% of the population. For iron, probability of adequacy values from Institute of Medicine [33] were used as the distribution of iron requirement is skewed (S2 Table). Considering the low dietary haem iron with high phytate and fibre in the plant foods commonly consumed by young children, PA values for iron were adjusted for 5% bioavailability. In agreement with the International Zinc Nutrition Consultative Group (iZiNCG), the EAR for zinc was also adjusted for 15% bioavailability for unrefined cereals based diets [34]. Mean PA for each nutrient was calculated for breastfed children of 6–11 months (except for vitamin A, vitamin C, thiamine, riboflavin, niacin and vitamin B6 intakes as information on the EAR and distributions for these nutrients for this age group are not available), breastfed children of 12–23 months and non-breastfed children of 12–23 months. For breastfed and non-breastfed children of 12–23 months, the mean probability of adequacy (MPA) was calculated, computed as the average of the PA of the 11 nutrients.

*Optimised diet for non-breastfed children of 12–23 months*: Dietary intakes were used as input for linear programming to develop an optimised diet for non-breastfed children of 12–23 months. First, the dietary intake data was used to define the model input parameters. These parameters included: a list of non-condiment foods consumed by ≥5 of the non-breastfed children of 12–23 months; the serving size of each food defined as the median serving size for all children who consumed the food; and the minimum and maximum number of servings per week for each food group and sub-food group defined as the 5^th^ and 95^th^ percentiles, respectively. The maximum number of servings per individual food within a subgroup was estimated based on percentage of children consuming that food. An energy constraint was used to ensure the modelled diet provided the average energy requirement for children of 12–23 months, estimated using their mean body weight (as measured in the study) and the FAO/WHO/UNU algorithm for estimating energy requirements [35]. Thirteen key nutrients were considered in the Optifood analysis: total fat, total protein, iron, zinc, calcium, vitamin A, vitamin C, thiamine, riboflavin, niacin, vitamin B6, folate, and vitamin B12. The FAO/WHO Recommended Nutrient Intakes (RNIs), representing the amount of a nutrient that ensures the needs of nearly all the population (97.5%), were used for all nutrients [31], except zinc which was defined by iZiNCG’s RNI for unrefined cereal based diets [34]. For fat, the average requirement of 30% of total energy was used. For iron 5% bioavailability and for zinc 15% bioavailability was assumed (as described above). Second, Optifood linear programming software (version 4.0.9, e-Optifood) was used to generate diets that best cover the nutrient needs of the target population. Nutrient intakes above 70% of RNI were classified as adequate, for most nutrients this represents at least the EAR, and it allows for comparison with other studies [8,9,22].

#### Production of households

The head of household of the selected child was interviewed to collect information on all crops cultivated during the previous year and the total production of the crop in local units together with the quantity used for home consumption, sold and/or other uses. Conversion factors were collected to convert local units to kg. The household crop production data was used to compute two measures of household crop diversity, both for total household production and quantity of household production used for home consumption. A simple crop count variable, used in previous studies to assess farm biodiversity [36,37], was computed by the sum of the total number of different crops cultivated by a household during the previous year. We quantified household crop production diversity using the Shannon-Wiener index that combines richness (number of crops) and evenness (distribution of quantity of production of different crops). The Shannon-Wiener index is defined as *H’* = -∑ (*p*_i_ log(*p*_i_)), where *p*_i_ is the relative abundance of occurrence of the *i*th crop produced by the household calculated as the proportion of the quantity of the *i*th crop to the total quantity of crops produced (total crop yield).

#### Food prices

A market survey was conducted to determine the price per edible 100g portion of all foods consumed by the children as identified in the 24hR. Enumerators bought food from food sellers within the communities visited and in the main markets within the research area. Each food was bought from three different food sellers and the price per 100 g edible portion from each seller was determined. For each food an average of the three prices were recorded as the price per 100 g edible portion. The average price per 100 g edible portion was used in converting monetary values of foods given during the 24hR to their weight equivalents and was used together with the total household crop production data (corrected for waste factors) to estimate total farm income and monetary value of total foods needed in the household.

#### Food and nutrients coverage of households

A household roster was filled including information for all individual household members on sex, age and physiological state (menstruation, pregnancy, lactating). The household composition data was used to calculate the total optimised food needs and nutrient needs of a household. For children below 23 months old we adjusted their nutrient needs by subtracting the nutrient intakes from average breastmilk intakes [29,30], as these nutrients do not need to be supplied by food. We assumed all children below 23 months old were breastfed [38].

The food coverage of a household was defined by the coverage of their food and food group needs from an optimised diet by their production. The optimised diet for non-breast children of 12–23 months was used to estimate the optimised food needs for all household members. Dietary patterns of this group were assumed to best resemble the food consumed in the household as most members do not consume breastmilk. Although not all foods consumed by adults might be given to young children [39] it was found that generally the diets of children after one year of age are integrated into family diets in our study location [40]. First, based on the household composition data, each household member was given a consumer unit respective to their age, sex and physiological state. We calculated consumer units for the different groups (by age, sex and physiological) by using their respective EARs of each of the 11 key nutrients relative to the EARs of women 19–50 years who are not pregnant or lactating (consumer unit is set to 1). For each group, an average was calculated of all these 11 consumer units of all key nutrients (S3 Table). For a child of 12–23 months the consumer unit was determined at 0.5. We used this approach to assure nutrient needs of all household members were more or less covered by the optimised diet. Second, the optimised food needs of a 12–23 months old child were doubled to arrive at the total foods needed for 1 consumer unit. For each household the consumer units were summed and these were multiplied by the optimised foods needed for one consumer unit to arrive at total household food and food group needs in kg per year. Third, the food coverage of a household was computed as the proportion of the foods and food groups produced by the household compared with the foods and food groups needed when adopting the FBDGs. Food groups were defined as in Optifood and foods and food groups were included if they were both recommended in the optimised diet and produced by households. Median household food coverage was calculated and the percentage of households above 100% food coverage at food and food group level. In addition, the proportion of households covering 100% or more of a specific number of food(s) (0 to 6) and food group(s) (0 to 3) was calculated. Similarly, these measures were also computed for the production of a household that was specifically reported to be used for home consumption. Assuming that the income from the foods produced is used to purchase other foods, the food coverage of a household in monetary value was calculated as the proportion of the monetary value of their production compared with the monetary value of their food needs. Median household food coverage based on monetary value was calculated as well as the percentage of households above 100% food coverage.

The nutrient coverage of a household was defined by the coverage of their nutrient needs by their production. The total energy and nutrient needs per household were calculated as the sum of the energy and nutrient needs per household member with the use of the household composition data together with the individual RNIs. The energy and nutrients supplied by the production of a household was calculated using the sFCT, that include adjustments for nutrient losses during cooking as described above but not for other post-harvest losses. For each household, the coverage of each nutrient was calculated as the proportion of the total quantity of nutrient produced and the total quantity of the nutrient needed. All individual nutrient coverages were truncated at 100%. Median household energy and nutrient coverages and the percentage of households below 70% of energy and nutrient coverage were calculated. The average coverage of all macro- and micro-nutrients was calculated. Similarly, these measures were also computed for nutrients supplied by the production of a household that was specifically reported to be used for home consumption.

#### Food coverage at household, regional and national level

For the household level, as described above, we calculated the median household food group coverage. For the district level, mean household food group coverage was calculated. As the mean also includes extreme values, it represents the potential of the district to cover the district’s food group needs. For an estimation of food group coverage at national level, the recommended total kg per food group per capita was compared with the total kg per food group available per capita per year, using the methodology of Keats and Wiggins [2]. As Ghana and other West African countries have no (or not sufficiently specific for this analysis) national FBDGs [7,41], the South African FBDGs [42] were used to calculate the recommended kg per food group per capita per year. The total food available per food group per capita per year was calculated from most recent data available from 2011 from the Food Balance Sheets accounting for food imports, exports and waste [43]. The quantity of different foods available per food group were summed and foods were included as was described by the South African guidelines.

### Statistical analysis

Statistical analyses were performed using SPSS (IBM SPSS Statistics 22) and R version 3.5.0 (R Core Team 2018). Data were checked for normality by visual inspection of histograms and Q-Q plots. Differences in the food and nutrient coverage of a household between the total quantity of their production and the total quantity of their production used for home consumption was analysed using Wilcoxon signed rank sum test (for continuous data) and McNemar Chi-square test (for categorical data). Differences in PA of 11 key nutrients and MPA of these nutrients between breastfed children of 12–23 months and non-breastfed of 12–23 months were analysed with Wilcoxon-Mann-Whitney test. The effects on the nutrition outcomes for a household (food and nutrient coverage) and a child(MPA and IDDS) of the diversity of the production of a household (crop count and Shannon-Wiener index), of the food coverage of a household (no. of food groups covered and overall coverage in GHS) and of the nutrient coverage of a household (% micronutrients covered and % macronutrients covered) were estimated using linear mixed models, taking location as a random factor (nested within main independent variable of specific model) and socio-economic and demographic household characteristics as fixed factors in the model to control influences of these characteristics. A recent review shows socio-economic factors are related with dietary patterns in LMICS [44]. The effect of count-dependent variables was estimated using Poisson regression models (no. of food groups covered) and a quasi-binomial regression models (IDDS). *P* value <0.05 was regarded as statistically significant.

### Ethical considerations

Clearance to carry out the research was granted by the Noguchi Memorial Institute for Medical Research Institutional Review Board (Ethical Clearance certificate No. NMIMR-IRB CPN 087/13-14). Approval for the study was obtained by the District Assembly, District Health Administration in Karaga and leaders of selected communities. Participation was voluntary and written informed consent was obtained from caregivers of selected children and thumb prints used for those who were not literate. The identity of the infants and their mothers/caregivers has been kept confidential. Caregivers were compensated with a 500 g sachet of iodized salt for their time.

## Results

### Characteristics of the study population

**Fig 2:**
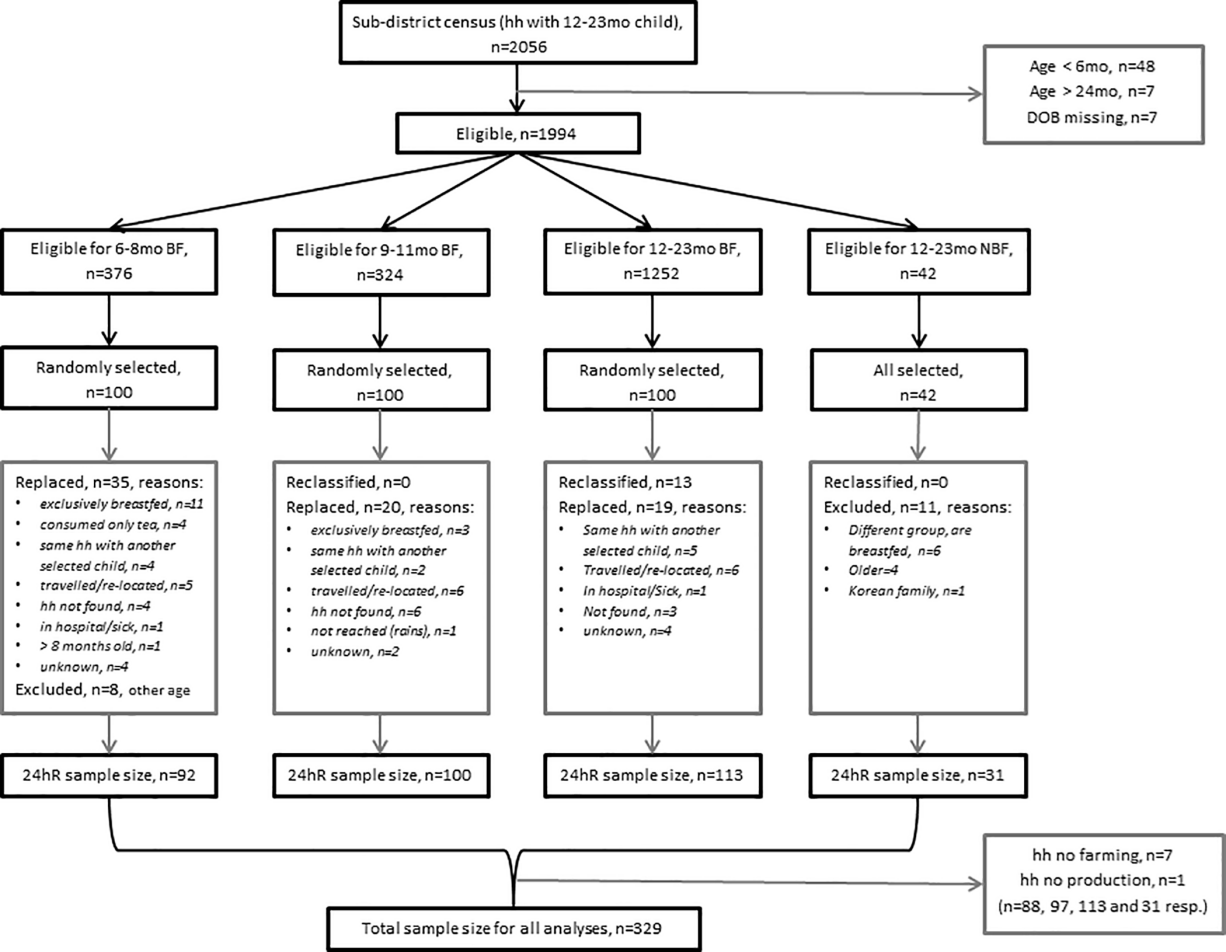
Flow chart of sample selection. hh = household. *n* = sample size. BF = breastfed. NBF = non-breastfed. Reclassified = from other age group to this group (different age or breastfeeding status during 24hour recall than census). 24hR = 24hour recall.

In total 329 households were included in the study (Fig 2). The selected children in the households were on average 12 months old, with about half being female (Table 1). Of all children 40% were stunted, 13% wasted and 1 child was overweight. More than half of the children had an IDDS of 4 or higher, reflecting a nutrient adequate diet [26]. Information on the exact foods and their quantities consumed by our study population is published elsewhere [45]. On average the mean probability of adequacy (MPA) of 11 micronutrients was 52% for breastfed children of 6–11 months, 49% for breastfed children of 12–23 months and 50% for non-breastfed children of 12–23 months (Table 2). Only thiamine, riboflavin and vitamin B_6_ had a probability of adequacy (PA) of 50% or more in all three groups. The PA of vitamin A and vitamin C were greater among breastfed children of 12–23 months than among those that were not breastfed (65% vs 12% and 72% vs 8%, respectively, *P*<0.05) while adequacies of calcium, iron, zinc, thiamine, niacin, vitamin B_6_ and vitamin B_12_ intake were less. The majority of the mothers and heads of household had not completed any formal education (93% and 85%). Their main occupation was farming which was the source of most of their income. Almost all households had a male household head and were Muslim. Households consisted on average of 14 members, with 6 adults and 3 children below 5 years old. Travel distance to the closest market was on average 60 minutes. On average households cultivated 5 ha with four different crops of which three were used for home consumption. Most households produced grains (97%) and legumes, nuts and seeds (84%) but only 8% of households produced vegetables.

**Table 1:** Demographic and social economic characteristics of children aged 6 to 23 months, their mothers and head of household and their households (*n* = 329).

	*Median (IQR) or %*
**Children characteristics**	
Age (in months)^a^	11.6 (8.2)
Female, %	51.1
Stunted^b^, %	39.8
Wasted^b^, %	13.3
Overweight^b^, %	0.3
*Dietary diversity*	
IDDS^c^ (0–7)^a^	4 (4)
IDDS^d^ ≥ 4(min. dietary diversity)^b^, %	56.8
*% consuming food group*	
Grains, roots and tubers	96.4
Legumes and nuts	60.8
Dairy products	13.7
Flesh foods	60.8
Eggs	1.5
Vitamin A rich fruits and vegetables	49.8
Other fruits and vegetables	49.2
**Mother and head of hh**^**e**^ **characteristics**	
*Education level completed*, *mother/head of hh*^**e**^	
None, %	92.7/84.5
Primary education, %	3.6/8.8
Higher education, %	3.6/6.4
*Occupation*, *mother/head of hh*^**e**^	
Farmer	63.5/80.5
Trader	18.2/9.4
*Income*, *mother/head of hh*^**e**^^,^ ^*f*^	
None, %	19.1/6.1
Mainly farm income, %	59.3/75.4
Mainly off-farm income, %	21.0/18.2
More than 7 GHS/week^g^, %	15.5/31.0
**Household characteristics**	
Household size	14 (13)
Adults in household	6 (6)
Children <5 years in household	3 (3)
Female headed households, %	1.5
Muslim, %	90.3
Market distance^h^, reported in minutes	60 (75)
Total cultivated area (ha)	5 (6.5)
Total value of assets in hh^**e**^^,^ ^i^ (PPP US dollar)	1579 (1550)
*Crop diversity*, *total production/for consumption*	
Crop count (Richness)	4 (2)/3 (2)
Shannon-Wiener Index	1.0 (0.6)/0.8 (0.5)

^a^One missing value: date of birth, n = 328

^b^Two missing values: 1 date of birth and 1 anthropometry measurements, n = 327

^c^Individual dietary diversity score (IDDS)

^d^An IDDS of 4 or more in infants and young children reflect a nutrient adequate diet [26]

^e^hh = household

^f^Two missing values for mothers and one missing value for head of household

^g^Estimated to be above average income per capita in the study location

^h^15 missing values: 3 missing, 8 households not visit market and 4 households where the mother does not go to market, n = 314

^i^Summed value of functioning assets in the household using estimated local market prices, expressed in Purchasing Power Parity (PPP) in US dollar (1 Ghanaian cedi = 0.9690 PPP US dollar).

**Table 2:** Probability of adequacy of micronutrients of children’s current diet.

	Breastfed children 6–11 mo *(n = 185)*	Breastfed children 12–23 mo *(n = 113)*	Non-breastfed children 12–23 mo *(n = 31)*
Nutrients	*Mean % (95%CI)*		
Calcium	16.6 (12.6–20.6)	3.7 (0.5–6.8)	13.1 (1.2–25.1)*
Iron	1.9 (0.7–3.0)	15.0 (11.6–18.4)	46.5 (35.4–57.5)*
Zinc	13.3 (9.7–16.9)	80.5 (74.9–86.1)	95.5 (89.8–101.1)*
Vitamin A	NA	64.5 (61.1–68.0)	11.7 (0.4–22.9)*
Thiamine	NA	80.0 (73.6–86.4)	96.0 (89.4–102.6)*
Riboflavin	NA	54.2 (46.4–62.1)	65.1 (49.5–80.7)
Niacin	NA	50.2 (42.1–58.2)	75.3 (62.7–87.9)*
Vitamin B_6_	NA	72.8 (65.4–80.2)	92.9 (84.2–101.7)*
Folate	62.4 (57.5–67.2)	23.5 (16.7–30.2)	32.5 (17.1–47.9)*
Vitamin B_12_	84.5 (82.6–86.3)	24.8 (19.7–29.9)	13.3 (1.0–25.5)*
Vitamin C	NA	71.8 (65.9–77.6)	7.9 (0.0–16.6)*
*MPA*^*a*^	*NA*	*49*.*2 (44*.*9–53*.*4)*	*50*.*0 (43*.*3–56*.*6)*

*P <0.05, Wilcoxon-Mann-Whitney test comparing breastfed and non-breastfed children 12–23 months

^a^MPA = Mean Probability of Adequacy is a summary measure of nutrient adequacy based on calculated probability of adequacy for calcium, iron, zinc, vitamin A, thiamine, riboflavin, niacin, vitamin B_6_, folate, vitamin B_12_ and vitamin C based on their respective estimated average requirements (EAR) and distributions

### Best optimised diet

The best optimised diet for non-breastfed children of 12–23 months old (representing 0.5 consumer unit) includes on an annual basis 2.3 kg of fats (fortified vegetable oil), 42.9 kg of grains (mainly white maize flour), 21.8 kg of legumes, nuts and seeds (mainly cowpea (*Vigna unguiculata)* and groundnut paste (*Arachis hypogaea*)), 1.6 kg of meat, fish (smoked anchovies) and eggs, and 26.3 kg of vegetables (mainly okro (*Abelmoschus esculentus)*, and kenaf leaves (*Hibiscus cannabinus)*) (Table 3). The optimised diet covers less than 70% of the RNI of calcium (33%), vitamin A (30%), vitamin B_12_ (2%) and vitamin C (42%) (S4 Table). Converting this optimised diet to other household members using CUs resulted in deficits of the same nutrients. In addition to these problem nutrients, energy (65%), fat (57%), iron (31%) and folate (67%) were also below 70% of the RNI for women 19–50 years (1 CU). On average for all household members combined energy, fat and iron were also below 70% of the summed RNI of households (in % median (IQR): 67.7 (2.9), 60.4 (2.3) and 60.3 (13.0), respectively) (S4 Table).

**Table 3:** Best optimised local feasible diet for children not breastfed 12–23 months old: recommended servings per week, median servings size and recommended serving size per year.

Foods^a^ per food group	Servings /week	Median serving size (g)	Quantity (kg) /year
	*Children not breastfed 12–23 mo (0*.*5 CU*^*b*^*)*		
Added fats	7	6.4	2.3
Vegetable oil fortified			
Grains	2	66.4	6.9
Sorghum dough	1	38.4	2.0
Maize dough, white	4.3	125.0	28.0
Maize flour, white	1	11.0	0.6
Maize grain, dried white	1	102.7	5.4
Rice brown, unpolished			
Legumes, nuts & seeds	4	41.6	8.7
Cowpea, white dried	2	12.5	1.3
Groundnut flour	7	25.2	9.2
Groundnut roasted, paste	7	1.0	0.4
Groundnut shelled, dried	3	14.5	2.3
Melon seed, roasted			
Meat, fish and eggs	7	4.5	1.6
Anchovies, smoked			
Vegetables	4	18.3	3.8
Jute leaves	7	18.9	6.9
Kenaf leaves	5	5.7	1.5
Onion bulb	7	4.2	1.5
Okro fruit, dried	7	27.5	10.0
Okro fruit	5	9.7	2.5
Tomato paste			

^a^Scientific/local names for some of the foods are as following: sorghum (Sorghum bicolor), cowpea (Vigna unguiculata), groundnut (Arachis hypogaea), melon seed (Cucumis melo seeds/neri), jute leaves (Corchorus olitorious /ayoyo leaves), kenaf leaves (Hibiscus cannabinus/bra leaves) and okro (Abelmoschus esculentus/okro).

^b^CU = consumer unit.

### Coverage of the food and food group needs from an optimised local diet of a household by their production

Own food production allowed about 60% of households to cover their needs for maize and groundnut, less than 40% for rice and sorghum, and less than 5% for cowpea and okro. At food group level, including also other foods produced belonging to the same food groups, about 60% of households did cover their grain and legume needs but none covered their vegetables needs from their own production (Table 4). Most households covered one or two of their food group needs by their own production (40.7% and 41.3%, respectively) (Table 5). Comparison of the monetary value of all household foods needed with the value of all household foods produced, showed that 63.8% of households were able to cover their food needs while 36.2% were not even if they used all of their income from sales of their own crop production to purchase food (Table 4). Among these 36.2% of households, 65% neither the household head nor the mother had an off-farm income as their main source of income, suggesting that about 20% of all households were unable to cover their food needs from their own food production (either by direct consumption or via farm income) and/or off-farm income.

**Table 4:** Coverage of foods and food groups^ from an optimised diet of a household by their production.

% food coverage^a^		Total household production (n = 329)			Household production used for consumption (n = 328)	
	*No*. *of hh*^*b*^ producing crop		*Median*^a^ *(IQR)*	*% of hh*^*b*^ *> 100%*	*Median*^a^ *(IQR)*	*% of hh*^*b*^ *> 100%*
*Food level*^*c*^						
Maize	311	134 (206)		62.0	111 (145)*	54.3*
Rice	132	0 (607)		37.7	0 (103)*	25.6 *
Sorghum	1	0 (28)		18.2	0 (6)*	15.5*
Cowpea	24	0 (0)		3.3	0 (0)*	1.8
Groundnut	220	125 (416)		54.1	34 (100)*	25.0*
Okro	24	0 (0)		0.3	0 (0)*	0.0^
*Food group level*^*c*^^,^^*d*^						
Grains	318	150 (244)		61.4	96 (123)*	48.2*
Legumes	277	160 (291)		62.6	26 (71)*	17.7*
Beans	216	118 (344)		51.4	0 (31)*	11.9*
Nuts, seeds	220	103 (343)		50.2	28 (82)*	22.0*
Vegetables	25	0 (0)		0.3	0 (0)*	0.0^
*All foods in monetary value*^*e*^		138 (196)		63.8		

^Foods and food groups are defined as by Optifood and included if they are both recommended in the optimised diet and produced by households.

*P <0.05, Wilcoxon signed rank sum test (continuous data) and McNemar Chi-square test (categorical data)comparing total household own production and household own production used for consumption, ^McNemar Chi-square test was not computed because one of variable is constant for all cases.

^a^Quantity crop produced/quantity crop needed*100. Crop needs for children below 23 months are adjusted by subtracting the nutrient intakes from average breastmilk intakes.

^b^hh = household

^c^Sorghum flour, maize flour, okro fruit raw and dried, onion bulb, jute leaves and kenaf leaves quantities needed are corrected for waste

^d^Grains produced include sorghum, maize, rice, and millet. Legumes produced include Bambara groundnut, cowpea, pigeon pea, soybean (Glycine max) (food group: beans) and groundnut (Arachis hypogaea) (food group: nuts and seeds). Vegetables produced include okro, tomatoes and cucumber

^e^Total food production of a household in Ghanaian Cedi’s (potential farm income)/total value of foods needed in Ghanaian Cedi’s (GHS)*100.

**Table 5:** Coverage of foods and food groups from optimised diet of a household by their production.

No. of foods/ groups covered^a^	Total household production (n = 329)	Household production used for consumption (n = 328)
	*% of hh*^*b*^	*% of hh*^*b*^
*Food level*		
0	9.4	23.2
1	31.0	41.2
2	39.2	25.0
3	15.2	9.1
4	5.2	0.9
5–6	0.0	0.0
*Food group level*		
0	17.6	44.8
1	40.7	44.5
2	41.3	10.7
3	0.3	0.0

^a^Number of foods and number of food groups covered (≥100%) by households

^b^Percentage of households that cover a specific total number of foods and food groups.

### Coverage of energy and nutrient needs of a household by their production

Overall 62% of the total quantity of micronutrients required by households was covered by their production. Less than 50% of the households covered their quantity of calcium, vitamin A, vitamin B_12_ and vitamin C required by their own food production (<70% of RNI). Overall 89% of total macronutrient requirements were covered by the production of a household, only fat was short (74%). Less than 50% of households covered the quantity of nutrients required by the household for most nutrients from their own production they indicated was consumed (Table 6).

**Table 6:** Coverage of energy and nutrients needs of a household by their production.

	Total household production (n = 329)		Household production used for consumption (n = 328)	
% nutrient coverage^a^	*Median % (IQR)*	*% of hh*^*b*^ *>70%*	*Median % (IQR)*	*% of hh*^*b*^ *>70%*
Energy (kcal)	100 (40)	70.2	45 (51)*	29.6*
*Macronutrients*				
Protein (g)	100 (0)	88.1	78 (55)*	57.6*
Fat (g)	74 (75)	51.7	23 (30)*	11.6*
Carbohydrate (g)	100 (0)	88.1	100 (37)*	70.7*
*Micronutrients*				
Calcium (mg)	33 (55)	**24.3**	9 (12)*	1.2*
Iron (mg)	80 (58)	56.5	35 (44)*	20.4*
Zinc (mg)	100 (25)	78.4	52 (61)*	36.6*
Vitamin A (μg)	0 (2)	**0.0**	0 (0)*	**0.0**^
Thiamine (mg)	100 (0)	90.9	100 (30)*	75.0*
Riboflavin (mg)	74 (60)	52.6	31 (41)*	15.9*
Niacin (mg)	100 (17)	77.5	63 (67)*	45.7*
Vitamin B_6_ (mg)	100 (0)	87.5	89 (48)*	64.9*
Folate (μg)	89 (58)	59.3	26 (33)*	13.1*
Vitamin B_12_ (μg)	0 (0)	**0.0**	0 (0)	**0.0**^
Vitamin C (mg)	0 (51)	**22.5**	0 (15)*	4.0*
*Overall*				
Macronutrients^c^	89 (26)	81.8	65 (36)*	43.3*
Micronutrients^d^	62 (26)	**34.0**	38 (30)*	3.4*

*P <0.05, Wilcoxon signed rank sum test (continuous data) and McNemar Chi-square test (categorical data) comparing total household own production and household own production used for consumption

^McNemar Chi-square test was not computed because one of variable is constant for all cases.

**Bold** = values that are less than 50% of households covered 70% of RNI of a specific nutrient.

^a^Quantity of nutrient produced/quantity nutrient needed*100. Nutrient needs for children below 23 months are adjusted by subtracting the nutrient intakes from average breastmilk intakes. Values at 100% cover the nutrient requirements per household per day (values higher than 100 percent are truncated to 100). Compared with recommended nutrient intakes (RNI), except for energy (energy requirements), protein (safe level), fat (total fat in grams), carbohydrates (Recommended Daily Allowance) and vitamin A (mean requirements).

^b^hh = household

^c^Macronutrients covered = average coverage of all macronutrients (protein, fat and carbohydrates)

^d^Micronutrients covered = average coverage of all 11 key micronutrients (calcium, iron, zinc, vitamin A, thiamine, riboflavin, niacin, vitamin B_6_, folate, vitamin B_12_ and vitamin C)

### The diversity of the production of households, the food and nutrient coverage of households and the children’s dietary diversity and nutrient adequacy

The diversity of the production of households was positively related with their food and nutrient coverage as well as the food coverage of households with their nutrient coverage. An increase of 1 unit of the Shannon-Wiener index resulted in households having 173 GH₵ extra value of foods produced to cover their needs. As maize costs 2,40 per kg, this means a households is able to buy 72 kg of extra maize during a year and with an average household size of 14 members it can cover 14 gram extra maize of the 168 grams needed by 1 CU per day. The diversity of the production of households, and the food and nutrient coverage of households were not related to their children’s dietary diversity and nutrient adequacy. Results were similar for the total production of households and their production used for home consumption except for the latter where crop count was positively related with children’s IDDS (Table 7). Among the households that did not fully cover their food needs by their own production estimated in monetary value, we also tested whether having off-farm income was associated with better nutrient adequate diets for children. The households where the mother and/or head of household reported they earned income off-farm did not have children with more nutrient adequate diets than households who did not (IDDS of 3.3(1.8) versus 3.6(1.8) and MPA of 52.6(23.3) versus 50.0(20.5), both *P*-value >0.05).

**Table 7:** Associations between the diversity of the production of households, the food and nutrient coverage of households and the children’s diet (*n* = 329), using linear mixed models.

	Household food coverage		Household nutrient coverage (RNI)		Children’s diet	
	Food groups^a^ (0–3)	All foods covered in GH₵^b^ (%)	Micro-nutrients covered^c^ (%)	Macro-nutrients covered^d^ (%)	MPA^e^ (%)	IDDS^f^ (0–7)
	*unstandardised Beta*					
*Total production of households (n = 329)*						
**Production diversity**						
Crop count^g^	0.1*	53.7*	6.4*	6.2*	0.00	0.02
Shannon-Wiener Index^h^	0.7*	172.9*	23.4*	26.4*	-0.05	-0.04
**Food coverage**						
Food groups covered^a^ (0–3)			19.8*	22.6*	-0.01	-0.20*
All foods covered in GH₵^b^ (%)			0.1	0.1	0.00	0.00
**Nutrient coverage**						
Micronutrients covered^c^ (%)					0.00	0.00
Macronutrients covered^d^ (%)					0.00	0.00
*Production for home consumption of households (n = 328)*						
**Production diversity**						
Crop count^g^	0.1*		6.1*	7.7*	0.00	0.79*
Shannon-Wiener Index^h^	0.3*		18.5*	23.9*	-0.02	1.08
**Food coverage**						
Food groups covered^a^ (0–3)			20.8*	28.5*	-0.03	-0.70
**Nutrient coverage**						
Micronutrients covered^c^ (%)					0.00	-0.04
Macronutrients covered^d^ (%)					0.00	-0.05

*P<0.05. Corrected for: household size, age household head and wife of household head, education household head and wife of household head, total household cropped area, household market distance, total value of household assets and random effect of location (nested within main independent fixed factor of specific model). For count dependent variable ‘Food group’ a Poisson regression was modelled, for ‘IDDS a quasi-binomial regression.

^a^Food groups covered = total number of food groups in a household that quantity needed is covered by household own production (grains, legumes and/or vegetables)

^b^All foods covered (GH₵) = total own production in GH₵ (potential farm income)/total value of foods needed in GH₵*100

^c^Micronutrients covered = average coverage of all 11 key micronutrients (calcium, iron, zinc, vitamin A, thiamine, riboflavin, niacin, vitamin B_6_, folate, vitamin B_12_ and vitamin C)

^d^Macronutrients covered = average coverage of all macronutrients (protein, fat and carbohydrates)

^e^MPA = Mean Probability of Adequacy is a summary measure of nutrient adequacy based on calculated probability of adequacy for calcium, iron, zinc, vitamin A, thiamine, riboflavin, niacin, vitamin B_6_, folate, vitamin B_12_ and vitamin C based on their respective estimated average requirements (EAR) and distributions. Only for children 12–23 months old.

^f^Individual dietary diversity score (IDDS) is computed by sum of seven food groups being consumed: 1. Grains, roots and tubers, 2. Legumes, nuts and seeds, 3. Dairy products, 4. Flesh foods, 5. Eggs, 6. Vitamin A rich fruits and vegetables and 7. Other fruits and vegetables [26]

^g^Crop count = the sum of the total number of different crops cultivated in a household during the previous year.

^h^Shannon-Wiener Index = combines richness (number of crops) and evenness (distribution of quantity of production of different crops)

### Comparison of food group coverage at household, district and national level

**Fig 3:**
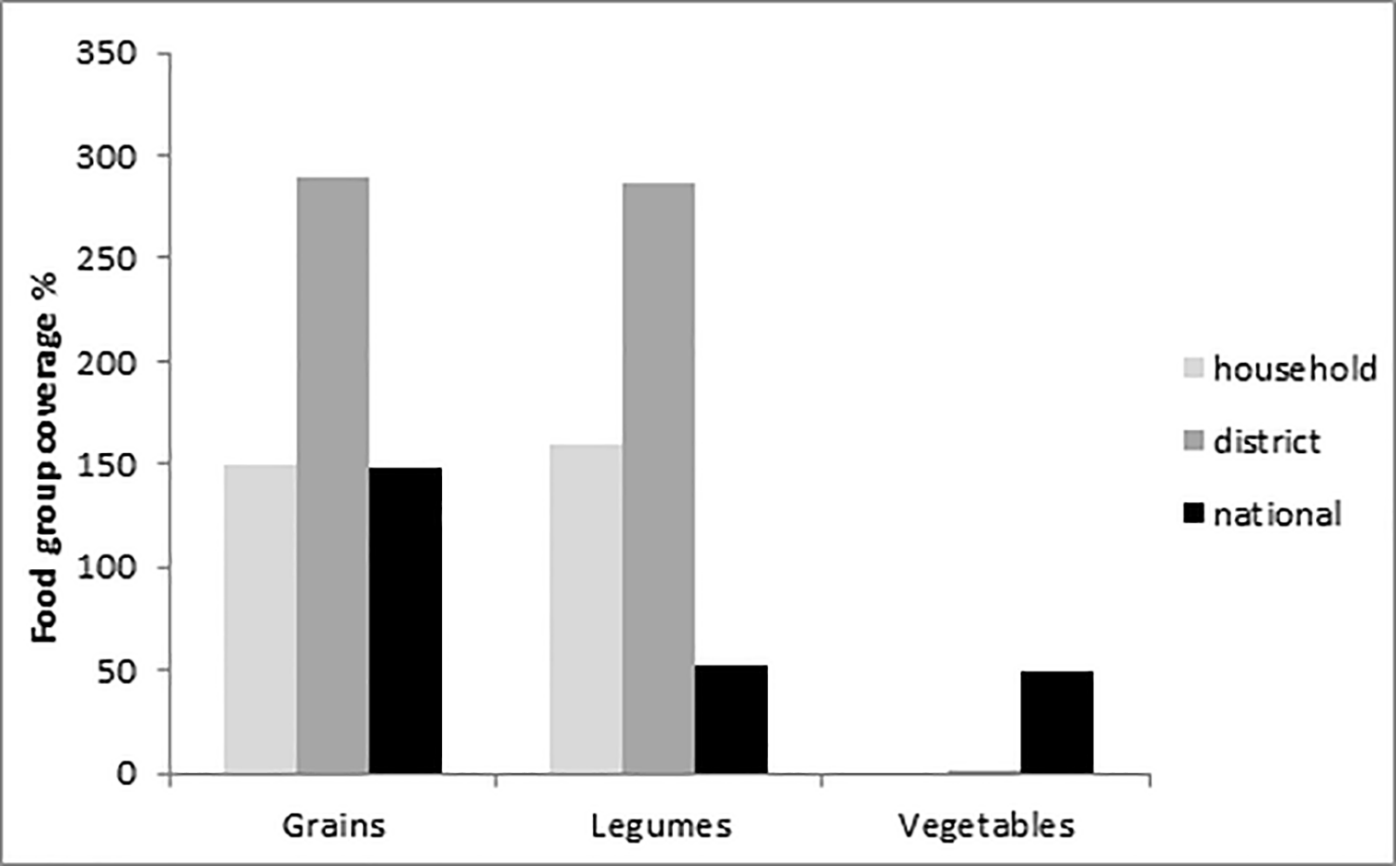
Coverage food groups at household, district and national level. Values at household level are in median (IQR); values at district level are mean (SD) based on household means from study population representing district coverage potential; and values at national level are percentages coverage (kg national food availability per capita/recommended food per capita (South African food-based dietary guidelines)*100). The grains food group at the national level also includes starchy roots (the South African guidelines does not include separate recommendations) which was not included at household and district level.

The food groups grains, legumes and vegetables were included as these were included in the optimised diet. Grain needs were amply covered by the production of households or national food availability (accounting for imports, exports and waste) at household (150%), district (267%) and national level (148%). At household and district level legume needs were also amply covered by production (160% and 268%, respectively) but not at national level (52%). At all levels, vegetable needs were not covered by the production of households or national food availability: at household and district level vegetable coverage was only 0% and 2% and at national level 49% (Fig 3).

## Discussion

The availability of recommended foods is a key condition for the adoption of food based dietary guidelines (FBDGs). We found that the production of households only partly covered the quantity and diversity of foods recommended by FBDGs and the nutrients required for all household members. Whereas the diversity of the production of households was positively associated with their food and nutrient coverage, there was no relationship with their child’s dietary diversity and nutrient adequacy.

### Scope of the study

Before discussing the results in detail, it is important to consider the scope of our study. First, although we sampled all non-breastfed children of 12–23 months in the district, our FBDGs are modelled based on dietary intake data from a relatively small sample size of 31 children as the vast majority of children of this age were breastfed. As we do not have dietary intake data from other household members (non- breastfed), the FBGDs from the non-breastfed children of 12–23 months were used to estimate optimised food needs for all household members. Dietary patterns may differ between young children and adults: in Ghana not all foods consumed by mothers were also given to young children [39]. In addition, out of home consumption is probably more common among older household members [46], indicating that we might overestimate the reliance on own production. Therefore we probably underestimate the variety of foods consumed by households and our results reflect a worst-case scenario. However, in general the diets of children older than one year are integrated into family diets in our study location [40] suggesting that the diets of young children are similar to that of adults. The possibility remains that households to which the non-breastfed 12–23 months old children belong may differ from other households with 12–23 months olds in the district as only few households had children in this age group who were not breastfed. However, we found no differences in household characteristics such as education, occupation and household size. In addition, the age of children in the non-breastfed group is higher compared to breastfed children in the same age group (mean of 21 months versus 17 months). The recent Ghanaian Demographic Health Survey also found a decrease in breastfed children with 91% of children being breastfed at age 12–17 months while 50% at age 20–23 months old [38]. This suggests that towards the age of two, less children are being breastfed in the study location and not that households necessarily differ in their beliefs and practices of feeding their younger children. Second, we used consumer units to translate the food needs according to the FBDGs for non-breastfed children of 12–23 months to the food needs of other household members. The consumer units were based on the average of estimated average requirements (EARs) of 11 key micronutrients relative to the EARS of women of reproductive age. However, individual nutrient needs differ for groups according to age, sex and physiological state. For example, pregnant women have a greater need of iron. However, when translating the optimised food needs of non-breastfed children of 12–23 months to food needs at household level, on average similar nutrients were below 70% of RNI (S4 Table). Third, dietary intake data was collected during one period of the year and cannot necessarily be extrapolated to other periods. Data was collected at the start of the rainy season (July 2014), also referred to as the ‘hunger season’ as this is the period of longest time from the previous harvest when crops are in the field but not yet producing food. The timing of the study was specifically chosen to coincide with the period of greatest food deficits. This may affect both children’s dietary intake data and food price data. Most children in our study did not consume fruits and only little vegetables. Seasonal variations in consumption of fruits, legumes, roots and plantains was reported among preschool children in Ghana and Malawi [47]. A recent study also found differences in dietary diversity among school children between the dry and rainy season in Northern Ghana, especially less vitamin A-rich fruits and vegetables were consumed during the dry season [48]. Conducting the study later in the rainy season could have resulted in larger fruit and more vegetable intakes, and therefore in FBDGs that better cover vitamin A and vitamin C requirements but also result in larger nutrient and food gaps. Food prices also tend to fluctuate during the year with prices depressed around harvest and highest prices during the ‘hunger season’. Due to urgent cash needs farmers tend to sell their surplus harvest and then end up buying food to cover the shortfall of foods at a time when prices are high [49]. Therefore the total monetary value of households own food production might be overestimated while the cost of food needs is probably not. When comparing monetary value of households own food production and monetary value of foods needs we might overestimate the coverage of households food needs by household own food production. Ideally, we should have collected dietary intake and food price data at least during two seasons, both hunger and harvest season. Fourth, our results depend on the quality of dietary recall data, the food composition data, assumed bioavailability of nutrients and RNI used. We used a multiple-pass procedure [25] to minimize bias in our dietary intake data. For collecting data on the production of households of the previous year, we also used a recall-based approach prone to systematic recall bias of foods and quantities of foods produced as well. Consumption of fruits and vegetables is often underestimated, especially of fruits that are mainly consumed as a snack [50,51], and fruits and vegetables are mostly cultivated in small quantities and often underreported. In addition, we did not include data on livestock that may also be available in the household and may have underestimated food diversity and especially the farm income of households as small-scale livestock rearing serves mostly as safety net to quickly access cash for emergency (medical) or planned expenditures (school fees) in Northern Ghana [52]. However, as these are mostly non-food expenditures, not including livestock will probably not have a major effect on our estimation of diversity of foods available in the household. Besides, the effect on our estimated nutrient gaps will also be limited as the consumption of animal sourced foods was extremely low in our study location.

### Current diets and FBDGs

We found that 40% of rural Northern Ghanaian infants and young children were stunted and their nutrient intakes were far below the required quantities: the probability of adequacy for most nutrient intakes was below 50%. This confirms the low quality diet and the need for FBDGs. Yet the FBDGs developed for non-breastfed children of 12–23 months using Optifood were unable to cover their calcium, vitamin A, vitamin B_12_ and vitamin C requirements. Their diet contained little if any animal-sourced foods (resulting in low calcium, vitamin A and vitamin B_12_ intakes), nor fresh fruits and vegetables (resulting in low vitamin A and vitamin C intakes) [32], as is typical for average diets of LIMC populations [2]. A similar dietary pattern was also found among school age children in Northern Ghana [48], as well as probabilities of adequacy of 0% for calcium, vitamin A, vitamin B_12_ and vitamin C intake among schoolchildren not receiving school feeding [53]. In line with our results, only 56% of children of 6–59 months consumed vitamin A rich foods in Northern Ghana [38] and 75.8% of children under 5 years were deficient in vitamin A [54]. Calcium, vitamin B_12_ and vitamin C are often neglected as key micronutrients due to the lack of strong evidence of direct associations of deficiencies with adverse health outcomes [32]. The addition of vitamin C to a meal enhances the absorption of non-haem iron and therefore a low vitamin C intake may exacerbate iron deficiency, especially when diets contain few animal-sourced foods. In Ghana, 82.1% of children of 6–59 months in Northern Ghana are anaemic (haemoglobin < 110 g/L) and one of the most common causes in Ghana is inadequate dietary intake of iron [38]. However, surprisingly, the optimised diet was able to cover iron and also zinc intakes for non-breastfed children of 12–23 months (not for children of 6–8 months, 9–11 months and 12–23 months receiving breastmilk), often identified as being difficult to cover for young children [29]. Maize and cowpea mostly contributed to both iron and zinc intakes, and green leafy vegetables to iron intake and brown rice to zinc intake. Overall in Ghana, the prevalence of anaemia decreases with increasing age of children although is still prevalent among older children [38]. As zinc deficiency is associated with stunting [55], and stunting levels are high among our study population, and often multiple micronutrient deficiencies coexist, it is likely that zinc deficiency is also common among children in Ghana [56].

### Nutrient and food gaps

The FBDGs developed in our study were based on extremes of the distribution of the types of foods consumed and on frequencies to arrive at FBDGs that cover most of the nutrient needs of our target group. Therefore, barriers in the food environment to adopt our FBDGs, such as lack of food accessibility, desirability and availability in households may exist. This was indicated by the high prevalence of iron and probably of zinc deficiency in Ghana despite the ability of the FBDGs to cover iron and zinc needs. Also, we found that for more than half of the households their own food production could allow to cover most of their micronutrient needs except for calcium, vitamin A, vitamin B12 and vitamin C. For other micronutrients not all households covered their needs, for example 43.5% of households were unable to cover their iron needs and 31.6% their zinc needs with their own production. This suggests that foods rich in specific nutrients have to be acquired through market, and in case of low (farm and off-farm) income, this may limit the intake of these nutrients and in turn limit the adoption of FBDGs. For successful adoption of these FBDGs, sufficient quantities of the recommended foods need to be available. About 60% of households produced sufficient grains and legumes themselves to cover their own needs. At district level both grain and legume production exceeded the requirements of the population within the district, yet this does not necessarily mean that FBDGs can be adopted by all households. To attain an adequate distribution of the grains and legumes produced to cover the needs of all individual households, regional and district markets need to function well and the farm income of households should be sufficient. The majority of households (97.5%) in our study population accessed local markets although their investment costs and time to do so varied. Unfortunately, we have no specific information on the quantity and diversity of foods available on these local markets. Maize (the main grain produced locally) is mostly grown for consumption, groundnuts and cowpea are partly grown for consumption and partly for sale, while soybean is mainly grown for sale and rarely consumed [57]. Although total legume production exceeds the district’s needs, there may be insufficient legumes available for purchase from local markets. A proportion of cowpea and groundnut is traded (half of households grow groundnuts for both home consumption and cash in Northern region [58]) through the main regional market in Tamale whereas all of the soybean is exported from the region to meet the national demand for livestock feed. In addition, Dillon and Barrett [59] found that generally sub-Saharan Africa has imperfect markets. Thus although production exceeds the district’s needs, legume crops might insufficiently be available for purchase from local markets since legumes are partly treated as cash crops. Nevertheless, the sale of the produce of households will contribute to their ability to buy foods that are available on the market. For 36.2% of households their overall farm income, measured as the total monetary value of their own crop production, was insufficient to cover the costs of their food needs. However, in 35% of these households either the household head or the mother or both had their main source of income off-farm that may be used to buy food to cover their needs. Yet this was not the case for the remaining 65% of these households. This suggests that overall about 20% of all households were unable to cover their food needs as they did not produce enough food and also lack other off-farm income sources. However, we have no information on the actual level of total off-farm income of households, as well as on other sources of food such as gifts, in kind, livestock and/or wild foods. Generally smallholder farmers in sub-Saharan Africa have other activities besides crop production, especially better-off smallholders achieve successful livelihood diversification [60]. Nevertheless, as for most rural households in Northern Ghana farm income is still the main source of income [14], our results suggest that for more than half of the households their own food production is sufficient to cover their food needs. However, besides assuming well-functioning markets, this also assumes that all available income would be used to purchase the quantities and diversity needed to fulfil the dietary needs of households, an assumption that most likely rarely holds [11,18]. At national level, grain production currently exceeds food needs but legume production does not. A recent analysis showed opposite results for grains but needs were compared with own production only and did not include, for example, rice imports [61]. Vegetables needs were not covered at household, district and national level. Together with fruits, there is often a shortage of vegetables in LMICs [2,62]. Further, compared with commodity crops like cereals, oilseeds and livestock, investment in agricultural research on vegetables in developing countries is limited [63]. The restricted availability of vegetables limits adoption of FBDGs.

### Diversifying crop production

Overall our study results show that the production of households partly supports the adoption of FBDGs in rural Northern Ghana. Diversifying crop production is often mentioned as a potential solution for increasing the diversity of foods available and thereby increasing dietary diversity of rural LIMC populations. Two recent reviews, of studies mostly conducted in sub-Saharan Africa suggests that agricultural biodiversity has a consistent association with more diverse diets at household and individual level [18,19]. However, the magnitude of the association is very small–African farms need to produce some nine additional species to increase dietary diversity by one food group [19]–and is stronger when current cropping system are less diverse[18,19]. We found that the diversity of the production of households was positively associated with their food and nutrient coverage but not with the quality of their children’s diet. To our knowledge, ours is the first study that included intermediate indicators such as the food and nutrient coverage of households: most other studies did not include validated IDDS for children 6 to 23 months old and/or quantitative dietary intake data (mean probability of adequacy). Our results suggest that increased diversity of the production of households does improve food and nutrient availability that may potentially cover the needs of the household. Farms with low crop biodiversity, as in our study are where households on average produce only four different crops, are associated with larger increases in dietary diversity when production is diversified than farms with already high crop biodiversity [18]. Nevertheless, we found no association with children’s diet, both their dietary diversity and the mean probability of nutrient adequacy of their diet. In the case of children’s dietary diversity this may be partly due to the fact that each food produced will add to households crop diversity regardless if they belong to the same food group while this is not the case if more foods from the same food group are consumed by children [64]. But we also do not find an association for the food and nutrient coverage of households with their children’s diet and for crop diversity with nutrient adequacy of their children’s diet. Overall these results are in line with what Sibhatu and Qaim [19] concluded from their quantitative meta-analysis, there is little evidence that increasing farm production diversity is a direct and effective strategy to improve smallholder diets and nutrition. They argue that further increasing production diversity in subsistence-oriented settings may maintain subsistence and reduce market opportunities. Therefore diversity at district scale may be more important in making sure that affordable diverse foods are available at local markets. This way rural households do not need to diversify their own production which may entail income losses through foregone gains from specialization [19]. Ecker [65] also concludes that in Ghana, where most regions undergo economic transformation, policies and programmes that support rural income growth may be more effective in improving dietary quality than those that promote farm production diversification. However, this depends on how income is spent. Another study conducted in the same location shows no improvements via the income pathway on children’s nutrition outcomes [21]. The role of markets need to be analysed in greater detail while studying the relation of farm production diversity and improving diets of rural LIMC populations.

### Implications and conclusion

Our study has several implications for future strategies to enhance rural diets and for research. First, as our FBDGs already show that with the existing local crops and the habitual dietary intakes certain nutrient requirements cannot be fulfilled, alternative options need to be considered. A recent study evaluating the implementation of FBDGs in Indonesia also shows that other strategies are needed to improve nutrient adequacy of vulnerable groups in addition to the adoption of FBDGs [66]. For example, strategies to enhance the productivity, production and/or consumption of foods rich in the nutrients that are in short supply (calcium, vitamin A, vitamin B_12_ and vitamin C) such as (dark green leafy) vegetables, beans, fruits and animal source foods. A recent randomized controlled trial in Burkina Faso showed that a homestead food production programme combined with a behaviour change communication programme significantly improved several child outcomes [67]. Nutrition-specific interventions like food fortification or supplementation are additional effective strategies to increase intake of these nutrients. As such, the national vitamin A supplementation program can significantly contribute to closing the Vitamin A gap, but coverage must be improved as only 44% of children of 6–59 months in Northern region in Ghana received supplementation [38].

Second, as we found that their own food production was not able to cover the food needs of many households, interventions are needed to increase the availability and/or accessibility of especially vegetables for all households and of grains and legumes for some households. Interventions increasing the production and/or improving productivity of these crops are needed in addition to interventions to promote the adoption of FBDGs. Besides production-oriented interventions, interventions that improve market accessibility of these foods may also be effective in covering the identified food gaps, assuming that households obtain sufficient farm and/or off-farm income to buy the quantity and diversity of foods needed and they are willing to spend their income as recommended. We found that most households sell part of their production, decreasing the food coverage at household level but increasing their farm income and potential food purchasing power. Therefore the availability of diverse foods at local markets, such as stimulation of vegetable production for local markets, may contribute to covering household food needs. However, market interventions are not easily implemented in remote settings and household production interventions may have higher short-term potential impact [68,69].

Our results show that although local FBDGs are based on actual dietary patterns and costs, the quantity and diversity of the production of households can limit their ability to adopt the FBDGs. Therefore, the promotion of food-based dietary guidelines through nutrition education or behavioural change communications activities alone is not enough to lead to improvements in diets. Additional strategies are required such as agricultural- and market-based strategies in combination with nutrition specific interventions such as food fortification and home fortification. These offer opportunities to further facilitate adoption of recommendations and provide additional support to improve diets of vulnerable populations.

## Supporting information

S1 Text Details on data collection and analysis. (DOCX)

S1 Table **Estimated average requirements (EAR) and distributions of zinc, calcium, vitamin A, vitamin C, thiamine, riboflavin, niacin, vitamin B**_**6**_**, folate, and vitamin B**_**12**_ **for children 6 to 12 months old (a) and children 1 to 3 years old (b).** RNI = Recommended nutrient intake. EAR = Estimated average requirements. Conversion = factor used to calculate the distribution. SD = standard deviation.
(DOCX)

S2 Table Probability of adequacy values for iron for children 6 to 12 months old and children 1 to 3 years old, assuming 5% bioavailability. (DOCX)

S3 Table Consumer units (CU) for translation of food-based dietary guidelines (FBDGs) for non-breastfed children 12 to 23months old to optimised food needs for all household members. Consumer units are based on EARs of each nutrient (WHO/FAO 2004) for specific group relative to EAR of women 19 to 50 years old who are not pregnant or lactating. Averages of the consumer units of all 11 nutrients were calculated for each group. Average consumer units are used to calculate quantity of foods needed for each household member based on FBDGs for non-breastfed children 12 to 23 months old.
(DOCX)

S4 Table Coverage of energy and nutrient requirements for children 12–23 months old, women 19 to 50 years old and at household level by the optimised diet. (DOCX)
