# Supplementary material for: Food and nutrient gaps in rural Northern Ghana: Does production of smallholder farming households support adoption of food-based dietary guidelines?
Source: PLoS One. 2018 Sep 13;13(9):e0204014. doi: 10.1371/journal.pone.0204014 (PMC6136797; doi:10.1371/journal.pone.0204014)
Supplement: S1 Text — (DOCX) [file pone.0204014.s001.docx]

## S1 Text. Details on data collection and analysis.

**Children’s nutritional status**

Weight was measured with an electronic scale to the nearest 0.1 kg (UNIscale: Seca GmbH, Hamburg, Germany). Length was measured with a UNICEF wooden three piece measuring board with a sliding foot piece and with a precision of 0.1 cm. Children were measured lying down. Both length and weight were measured twice for each child and the average of the two measurements was taken. Scales were calibrated with a standard weight at the start of each day of data collection. Age was calculated using the date of birth from verifiable documents (health record, weighing card, birth certificate) or estimated based on traditional calendar.

**Food composition table**

Where appropriate, yield (1) and nutrient retention factors (2, 3) were applied to account for nutrient losses during cooking. The Atwater general factors for carbohydrate, protein and fat and the recommended metabolizable energy for dietary fibre in ordinary diets (2 kcal or 8.4 kJ/g) are used in calculating energy (4). Total vitamin A (RAE) was calculated as the sum of retinol and 1/12 β-carotene (1).

**Children’s dietary intake**

Primary caretakers were asked to recall all the foods and drinks consumed in and outside the home by their child during the preceding day and to describe ingredients and cooking methods of any mixed dishes. Duplicate amounts of all foods and ingredients of mixed dishes consumed were weighed to the nearest 2g using Soehnle electronic kitchen scale (Plateau Art 65086, Germany). Scales were randomly assigned to the interviewers and calibrated with a known weight each day. When duplicates were not available in the household, amounts were estimated (in order of priority) as their monetary value equivalents, in weight-to-weight estimates with other foods (e.g. amount of sugar estimated with weight of same volume of corn flour), in volumes, as their general sizes (small, medium or large) using pictures or in household units. The total volume of each (mixed) dish cooked at the respondents’ household and the volume of this dish specifically consumed by the child were measured to determine the proportion of the dish consumed by the child. This proportion was multiplied by the total amount of ingredients used in the preparation of the dish to determine the amount of ingredients consumed by the child. Standard recipes were generated to estimate the grams of ingredients consumed from mixed dishes eaten outside the home by averaging three recipes of different vendors in the local area. Conversion factors were developed to convert monetary values, weight-to-weight measures, volumes, sizes and household units to their gram weight equivalents.

*Children’s nutrient adequacy:* Except for iron, the probability of adequacy (PA) of each nutrient was calculated based on their respective estimated average requirements (EARs) and distributions (44, 45) (S1 Table). The following formula was used in SPSS: PA=PROBNORM [(adjusted individual intake-EAR)/SD], where the PROBNORM function clarifies whether the probability of the individual intake is above the EAR. For iron, probability of adequacy values from Institute of Medicine (46) were used as the distribution of iron requirement is skewed (S2 Table).

*Optimised diet for non-breastfed children of 12-23 months:* The Optifood analysis comprises of four steps (5, 6) but for this study we only ran the first two steps: (1) to check that model parameters ensure realistic diets; and (2) to identify two realistic diets that meet or come as close as possible to meeting nutrient needs of the target population. One of the two modelled diets uses the median number of servings of foods (takes into account habitual food pattern: ‘food pattern diet’) while the other diet uses the extremes of the distributions (‘no food pattern diet’). We used the no food pattern diet for this study as this diet best covered requirements of all 13 key nutrients.

**Food coverage at household, regional and national level**

**The recommended and nationally supplied quantity of food groups per capita per year, and the percentage coverage of the recommended food groups by national food supply.**

| **Food group** | **Recommended SA^a^, kg/capita/year,**  **report** | **Supply^b^ kg/capita/year,**  **report** | **Coverage, %** |
| --- | --- | --- | --- |
| Starchy foods | 356 | 527 | **148.0** |
| Vegetables | 82 | 40 | **48.8** |
| Fruit | 55 | 172 | **312.7** |
| Dry beans, split peas, lentils, soya | 27 | 14 | **51.9** |
| Fish, chicken, lean meat, eggs | 37 | 47  *(27 from fish)* | **127.0** |
| Milk, maas (fermented milk), yoghurt | 55 | 9.4 | **17.1** |
| Fat, oil | 13 | 6.9 | **53.1** |
| Sugar (incl. sugar cane) | 13 | 16.5 | **126.9** |

*^a^Quantities as recommended by the South African food-based dietary guidelines, based on average for adult men and women
^b^Quantities based on most recent data available from 2011 from the Food Balance Sheets accounting for food imports, exports and waste*

**References**

1. FAO, INFOODS, ECOWAS/WAHO, Bioversity International. West African Food Composition Table. Rome, Italy: FAO; 2012.

2. USDA Nutrient Data Laboratory. USDA National Nutrient Database for Standard Reference, Release 28 2015. Version Current: September 2015:[Available from: <http://www.ars.usda.gov/nea/bhnrc/ndl>.

3. Vásquez-Caicedo AL, Bell S, Hartmann B. Report on collection of rules on use of recipe calculation procedures including the use of yield and retention factors for imputing nutrient values for composite foods (D2.2.9). EuroFIR. 2008.

4. FAO. Food energy – methods of analysis and conversion factors. Report of a technical workshop (FAO food and nutrition paper 77). Rome: Food and Agriculture Organisation. 2003.

5. Ferguson EL, Darmon N, Fahmida U, Fitriyanti S, Harper TB, Premachandra IM. Design of optimal food-based complementary feeding recommendations and identification of key "problem nutrients" using goal programming. The Journal of nutrition. 2006;136:2399-404.

6. Daelmans B, Ferguson E, Lutter CK, Singh N, Pachón H, Creed-Kanashiro H, et al. Designing appropriate complementary feeding recommendations: tools for programmatic action. Maternal & child nutrition. 2013;9 Suppl 2:116-30.
