# Supplementary material for: Food and nutrient gaps in rural Northern Ghana: Does production of smallholder farming households support adoption of food-based dietary guidelines?
Source: PLoS One. 2018 Sep 13;13(9):e0204014. doi: 10.1371/journal.pone.0204014 (PMC6136797; doi:10.1371/journal.pone.0204014)
Supplement: S1 Table — Estimated average requirements (EAR) and distributions of zinc, calcium, vitamin A, vitamin C, thiamine, riboflavin, niacin, vitamin B6, folate, and vitamin B12 for children 6 to 12 months old (a) and children 1 to 3 years old (b). RNI = Recommended nutrient intake. EAR = Estimated average requirements. Conversion = factor used to calculate the distribution. SD = standard deviation. (DOCX) [file pone.0204014.s002.docx]

## S1 Table. Estimated average requirements (EAR) to be used for assessing Probability of Adequacy, for infants 6 to 12 months and children 1 to 3 years old^a, b^

|  | **Infants 6 to 12 months old** | | | **Children 1 to 3 years old** | | |
| --- | --- | --- | --- | --- | --- | --- |
| **Nutrient** | RNI | **EAR** | **SD^c^** | RNI | **EAR** | **SD^d^** |
| Zinc (mg), low bioavailability | 5^e^ | 4^e^ | 0.5 | 3 | 2^e^ | 0.5 |
| Calcium (mg) | 400 | 300^f^ | 50 | 500 | 370^g^ | 64.75 |
| Vitamin A (µg) | 400 | n/a | n/a | 400 | 286^g^ | 57.2 |
| Vitamin C (mg) | 30 | n/a | n/a | 30 | 25^g^ | 2.5 |
| Thiamine (mg) | 0.3 | n/a | n/a | 0.5 | 0.42^g^ | 0.04 |
| Riboflavin (mg) | 0.4 | n/a | n/a | 0.5 | 0.42^g^ | 0.04 |
| Niacin (mg) | 4.0 | n/a | n/a | 6 | 4.6^g^ | 0.69 |
| Vitamin B_6_ (mg) | 0.3 | n/a | n/a | 0.5 | 0.42^g^ | 0.04 |
| Folate (µg) | 80 | 65^f^ | 7.48 | 150 | 120^f^ | 15 |
| Vitamin B_12_ (µg) | 0.7 | 0.6^f^ | 0.05 | 0.9 | 0.7^f^ | 0.105 |

RNI = Recommended nutrient intake. EAR = Estimated average requirements. SD = standard deviation. n/a = not available.  *^a^All values are taken from WHO/FAO (2004) unless otherwise stated.
^b^ Values for EAR are adjusted for an assumed bioavailability (WHO/FAO, 2004). Thus, EAR refers to intake of the nutrients and not the physiological need for the absorbed nutrient
^c^All SDs for infants 6 to 12 months were calculated based on RNI and EAR, using conversion factor RNI/EAR. If EARs were not available, SDs could not be calculated
^d^All SDs for children 1 to 3 years were calculated based on EAR and CV (SD=CV*EAR/100). CV is assumed to be 10% for all micronutrients except 15% for niacin (IOM, 2002), 20% for vitamin A (IOM, 2002), 17.5% for calcium (WHO/FAO 2004) and conversion for zinc, folate and vitamin B_12_ were calculated with RNI/EAR
^e^Values are taken from iZiNCG (2004)
^f^ EAR taken from WHO/FAO (2004)
^g^ EAR back-calculated from RNI (Recommended Nutrient Intake) (WHO/FAO, 2004)*
