# Supplementary material for: Food and nutrient gaps in rural Northern Ghana: Does production of smallholder farming households support adoption of food-based dietary guidelines?
Source: PLoS One. 2018 Sep 13;13(9):e0204014. doi: 10.1371/journal.pone.0204014 (PMC6136797; doi:10.1371/journal.pone.0204014)
Supplement: S2 Table — (DOCX) [file pone.0204014.s003.docx]

## S2 Table. Probability of adequacy values for iron for children 6 to 12 months old and children 1 to 3 years old, assuming 5% bioavailability.

| **Probability of adequacy** | **6-12 months,**  **10% bioavailability^a^** | **6-12 months,**  **total absorbed^b^** | **6-12 months,**  **5% bioavailability^c^** | **1-3 years,**  **5% bioavailability^d^** |
| --- | --- | --- | --- | --- |
| 0 | <3.01 | 0.301 | **<6.02** | **<3.64** |
| 0.04 | 3.02-3.63 | 0.302-0.363 | **6.03-7.26** | **3.65-4.46** |
| 0.07 | 3.64-4.35 | 0.364-0.435 | **7.27-8.70** | **4.47-5.54** |
| 0.15 | 4.36-5.23 | 0.436-0.523 | **8.71-10.46** | **5.55-7.06** |
| 0.25 | 5.24-5.87 | 0.524-0.587 | **10.47-11.74** | **7.07-8.35** |
| 0.35 | 5.88-6.39 | 0.588-0.639 | **11.75-12.78** | **8.36-9.58** |
| 0.45 | 6.40-6.90 | 0.640-0.690 | **13.80** | **9.59-10.84** |
| 0.55 | 6.91-7.41 | 0.691-0.741 | **14.82** | **10.85-12.20** |
| 0.65 | 7.42-7.93 | 0.742-0.793 | **15.86** | **12.21-13.75** |
| 0.75 | 7.94-8.57 | 0.794-0.857 | **17.14** | **13.76-15.80** |
| 0.85 | 8.58-9.44 | 0.858-0.944 | **18.88** | **15.81-18.94** |
| 0.92 | 9.45-10.15 | 0.945-1.025 | **20.50** | **18.95-21.82** |
| 0.96 | 10.16-10.78 | 1.016-1.078 | **21.56** | **21.83-24.52** |
| 1 | >10.78 | >1.078 | **>21.56** | **>24.52** |

*^a^Values from Tables 1-3 from IOM 2001
^b^Calculated total absorbed iron needed, assuming 10% bioavailability
^c^Calculated total absorbed iron needed, assuming 10% bioavailability (total absorbed needed when assuming 10% bioavailability*0.1/0.05)
^d^Values from WHO/FAO 2006*
