# Supplementary material for: Food and nutrient gaps in rural Northern Ghana: Does production of smallholder farming households support adoption of food-based dietary guidelines?
Source: PLoS One. 2018 Sep 13;13(9):e0204014. doi: 10.1371/journal.pone.0204014 (PMC6136797; doi:10.1371/journal.pone.0204014)
Supplement: S3 Table — Consumer units are based on EARs of each nutrient (WHO/FAO 2004) for specific group relative to EAR of women 19 to 50 years old who are not pregnant or lactating. Averages of the consumer units of all 11 nutrients were calculated for each group. Average consumer units are used to calculate quantity of foods needed for each household member based on FBDGs for non-breastfed children 12 to 23 months old. (DOCX) [file pone.0204014.s004.docx]

## S3 Table. Consumer units (CU) for translation of food-based dietary guidelines (FBDGs) for non-breastfed children 12 to 23months old to optimised food needs for all household members.

| **Groups WHO** | **Calcium** | **Zinc** | **Iron** | **Vitamin A** | **Thiamine** | **Riboflavin** | **Niacin** | **Vitamin B6** | **Folate** | **Vitamin B12** | **Vitamin C** | ***Average*** |
| --- | --- | --- | --- | --- | --- | --- | --- | --- | --- | --- | --- | --- |
|  | *Consumer Units (CU)* | | | | | | | | | | | |
| 0 - 6 months | 0.1 | **0.8** | 0 | 0 | 0.1 | 0.1 | 0.1 | 0 | 0 | 0 | 0 | *0.4* |
| 7 - 12 months | 0.1 | 0.5 | 0.5 | 0 | 0.2 | 0.2 | 0.3 | 0.2 | 0 | 0 | **0.7** | *0.5* |
| 1 - 3 months | 0.3 | 0.2 | 0.4 | 0 | 0.3 | 0.2 | 0.4 | 0.3 | 0.2 | 0.1 | 0.1 | *0.5* |
| 4 - 6 y | 0.6 | 0.6 | 0.5 | 0.7 | 0.5 | 0.5 | 0.6 | 0.4 | 0.5 | 0.5 | 0.7 | *0.6* |
| 7 - 9 y | 0.7 | 0.6 | 0.5 | 0.9 | 0.8 | 0.8 | 0.9 | 0.7 | 0.8 | 0.8 | 0.8 | *0.7* |
| Females, 10 - 14 y, pre-menarche | 1.3 | 1.0 | **0.6** | **1.4** | 1.0 | 1.0 | 1.1 | 0.9 | 1.0 | 1.0 | 1.0 | *1.0* |
| Females, 10 - 14 y, menarche | 1.3 | 1.0 | 0.7 | **1.4** | 1.0 | 1.0 | 1.1 | 0.9 | 1.0 | 1.0 | 1.0 | *1.0* |
| Females, 15 - 18 y | 1.3 | 1.3 | 1.0 | 1.4 | 1.0 | 1.0 | 1.1 | 0.9 | 1.0 | 1.0 | 1.0 | *1.1* |
| Males, 10 - 14 y | 1.3 | 1.0 | **0.7** | 1.4 | 1.2 | 1.1 | 1.1 | 1.0 | 1.0 | 1.0 | 1.0 | *1.1* |
| Males, 15 - 18 y | 1.3 | **1.6** | 0.9 | 1.4 | 1.2 | 1.1 | 1.1 | 1.0 | 1.0 | 1.0 | 1.0 | *1.1* |
| Females, 19 - 50 y, premenopausal | 1.0 | 1.0 | 1.0 | 1.0 | 1.0 | 1.0 | 1.0 | 1.0 | 1.0 | 1.0 | 1.0 | *1.0* |
| Females, 51 - 65 y, menopausal | 1.3 | 1.0 | **0.5** | 1.0 | 1.0 | 1.0 | 1.0 | 1.2 | 1.0 | 1.0 | 1.0 | *1.0* |
| Males, 19 - 50 y | 1.0 | **2.1** | **0.7** | 1.1 | 1.2 | 1.1 | 1.1 | 1.0 | 1.0 | 1.0 | 1.1 | *1.1* |
| Males, 51 - 65 y | 1.0 | **2.1** | **0.7** | 1.1 | 1.2 | 1.1 | 1.1 | 1.3 | 1.0 | 1.0 | 1.1 | *1.2* |
| Females, 65+ y | 1.3 | 1.0 | **0.5** | 1.1 | 1.0 | 1.0 | 1.0 | 1.2 | 1.0 | 1.0 | 1.1 | *1.0* |
| Males, 65+ y | 1.3 | **2.1** | **0.7** | 1.1 | 1.2 | 1.1 | 1.1 | 1.3 | 1.0 | 1.0 | 1.1 | *1.2* |
| Pregnant women | **1.2** | 1.7 | **3.8** | 1.4 | 1.3 | 1.3 | 1.3 | 1.5 | 1.6 | **1.1** | 1.3 | *1.6* |
| Lactating women | 1.0 | 1.3 | **0.7** | **1.7** | 1.5 | 1.4 | 1.2 | 1.5 | 1.4 | 1.2 | **1.7** | *1.3* |

Consumer units are based on EARs of each nutrient (WHO/FAO 2004) for specific group relative to EAR of women 19 to 50 years old who are not pregnant or lactating. Averages of the consumer units of all 11 nutrients were calculated for each group (average are calculated for each row). Average consumer units are used to calculate quantity of foods needed for each household member based on FBDGs for non-breastfed children 12 to 23 months old.
***Bold*** *= 0.4 or more CU difference of a specific nutrient with average CU for all nutrients for a specific group.*
