# Supplementary material for: Food and nutrient gaps in rural Northern Ghana: Does production of smallholder farming households support adoption of food-based dietary guidelines?
Source: PLoS One. 2018 Sep 13;13(9):e0204014. doi: 10.1371/journal.pone.0204014 (PMC6136797; doi:10.1371/journal.pone.0204014)
Supplement: S4 Table — (DOCX) [file pone.0204014.s005.docx]

## S4 Table. Coverage of energy and nutrient requirements for children 12-23 months old, women 19 to 50 years old and at household level by the optimised diet.

|  | **Children 12-23mo (0.5CU)** | **Women 19-50yrs (1CU)** | **All household members** |
| --- | --- | --- | --- |
| **Nutrients** | Coverage %, RNI | Coverage %, RNI | Median (IQR)  Coverage %, RNI |
| Energy (Kcal) | 107.4 | **64.9**^a^ | **67.7 (2.9)** |
| *Macronutrients* |  |  |  |
| Protein | 304.8 | 118.3^c^ | 100.0 (0.0)^f^ |
| Fat | 125.4 | **56.8**^b^ | **60.4 (2.3)** |
| Carbohydrates | **-** | 143.0^d^ | 100.0 (0.0)^f^ |
| *Micronutrients* |  |  |  |
| Calcium | **33.2** | **33.2** | **34.3 (2.4)** |
| Iron | 78.0 | **30.8** | **60.3 (13.0)** |
| Zinc | 150.7 | 100.4 | 85.7 (9.9) |
| Vitamin A | **30.2** | **48.2**^e^ | **39.1 (2.7)** |
| Thiamin | 142.7 | 129.6 | 100.0 (0.0)^f^ |
| Riboflavin | 98.6 | 89.6 | 88.4 (3.2) |
| Niacin | 168.4 | 144.3 | 100.0 (0.0)^f^ |
| Vitamin B6 | 153.1 | 117.7 | 100.0 (0.0)^f^ |
| Folate | 89.4 | **67.0** | 73.5 (2.2) |
| Vitamin B12 | **2.3** | **2.2** | **2.3 (0.1)** |
| Vitamin C | **42.1** | **56.0** | **54.6 (4.0)** |

***Bold*** *= coverage below 70% of RNI ^a^energy requirements WHO 2001, assume moderate activity ^b^WHO 2010 AMDR, based on energy requirements
^c^Safe level.
^d^Recommended Dietary Allowance.  ^e^Mean requirements.  ^f^constant, all households have a coverage of 100%.*
